# Effects of theophylline therapy on respiratory muscle strength in patients with prolonged mechanical ventilation: A retrospective cohort study

**DOI:** 10.1097/MD.0000000000013982

**Published:** 2019-01-11

**Authors:** Teng-Jen Yu, Yu-Chih Liu, Chien-Min Chu, Han-Chung Hu, Kuo-Chin Kao

**Affiliations:** a Department of Pulmonary and Critical Care Medicine, Chang-Gung Memorial Hospital, Keelung; b Department of Thoracic Medicine, Chang-Gung Memorial Hospital; c Department of Respiratory Therapy, Chang-Gung University, Taoyuan, Taiwan.

**Keywords:** prolonged mechanical ventilation, respiratory muscle, theophylline, VIDD

## Abstract

Mechanical ventilation may cause diaphragm weakness an effect termed ventilator-induced diaphragm dysfunction (VIDD). The prevalence of VIDD among patients receiving mechanical ventilation is very high, with the degree of diaphragmatic atrophy being associated with the length of mechanical ventilation. Theophylline is known to increase diaphragmatic contractility and reduce fatigue, so in this study, we evaluated the effect of theophylline in patients with prolonged mechanical ventilation.

Patients who depended on mechanical ventilation were included in the study. We compared the maximum inspiratory pressure (PImax) values, rapid shallow breathing index (RSBI) values, and successful weaning rates of theophylline-treated and non-theophylline-treated patients.

Eighty-four patients received theophylline and 76 patients did not. These 2 groups’ clinical characteristics, including their PImax and RSBI at initial admission, were similar. The results showed that the theophylline-treated group had significantly better PImax and RSBI, with a higher last PImax (30.1 ± 9.7 cmH_2_O vs 26.9 ± 9.1 cmH_2_O; *P* = .034) and lower last RSBI (107.0 ± 68.4 vs 131.4 ± 77.7; *P* = .036). The improvements to each respective patient's PImax and RSBI were also significantly higher in the theophylline-treated group (PImax: 20.1 ± 5.7% vs 3.2 ± 1.1%, *P* = .005; RSBI: 11.2 ± 3.0% vs 2.7 ± 1.6%, *P* = .015). The weaning success rate of the theophylline-treated group was also higher, but not significantly so.

Theophylline might improve respiratory muscle strength in patients with prolonged mechanical ventilation and it needs further prospective studies to confirm.

## 1 Introduction

Many patients admitted to intensive care units (ICUs) require mechanical ventilation to support respiration. However, mechanical ventilation itself may have harmful effects on the diaphragm. Specifically, it may induce diaphragmatic weakness and contractile dysfunction through muscle injury and atrophy, an effect termed ventilator-induced diaphragmatic dysfunction (VIDD).^[1,2]^ Diaphragmatic weakness, in turn, has been postulated to be a major contribution to difficult weaning from mechanical ventilation.^[3,4]^

Most previous studies of VIDD were performed on animals.^[5–7]^ Meanwhile, VIDD-related studies involving humans have been rare and have mostly involved patients in ICUs.^[8–10]^ According to the limited data currently available, the clinical management of VIDD may include optimizing the mode of mechanical ventilation in order to avoid prolonged complete rest and to preserve contractions of the diaphragm.^[11–13]^ In addition, respiratory muscle training may also have beneficial effects.^[14]^ In terms of pharmacological interventions, there is no medical treatment for VIDD available at present, although some investigations of medications are underway.^[15]^

Theophylline, a methylxanthine, is a muscle stimulant. It increases cardiac muscle contractibility and increases the endurance and strength of respiratory muscles, including the intercostal muscles and diaphragm.^[16–18]^ These characteristics of theophylline suggest that it may have beneficial effects in the management of VIDD. Recently, Kim *et al* reported a study of theophylline treatment conducted with ICU patients that included ultrasonographic examinations for the assessment of diaphragmatic motion; they found that theophylline significantly improved diaphragmatic movements in patients with VIDD.^[10]^ These findings further suggested that theophylline may be of therapeutic value to patients with mechanical ventilation weaning difficulties associated with VIDD. Therefore, in this study, we evaluated the effects of theophylline in a group of prolonged mechanical ventilation patients (defined as patients requiring mechanical ventilation for more than 21 days) who had weaning difficulties and should have had a high prevalence of VIDD due to long-term mechanical ventilation support.

## 2 Methods

### 2.1 Subjects

In Taiwan, National Health Insurance Bureau policy requires that patients who have stayed in an ICU for more than 21 days (ie, those requiring prolonged mechanical ventilation) but who no longer require ICU monitoring should be transferred to a respiratory care center, a center used to manage patients requiring specialized respiratory care. If such a patient still cannot be weaned from his or her ventilator after 42 days in the respiratory care center, the patient will then be transferred to a respiratory care ward. This study was a retrospective cohort study that reviewed the data of patients admitted to the respiratory care center at Chang Gung Memorial Hospital in Keelung, Taiwan, between March 2013 and October 2015. The exclusion criteria for the study were as follows:

1. death in the respiratory care center,
2. diaphragmatic palsy,
3. thoracostomy,
4. pneumothorax,
5. massive pleural effusion,
6. neuromuscular disease (eg, myasthenia gravis, Guillain-Barré syndrome, and amyotrophic lateral sclerosis),
7. uncontrolled seizure, and
8. liver cirrhosis or active hepatitis.

This study was approved by the Institutional Review Board of Chang Gung Memorial Hospital (approval number: 201601048B0) and the requirement for written informed consent was waived.

### 2.2 Study design

In our respiratory care center, for the period from July 2014 through October 2015 (ie, for 16 months), all the patients were treated with aminophylline 200 mg (which contains 85.7% of anhydrous theophylline) bid, unless contraindicated (ie, uncontrolled arrhythmia or seizure, and liver cirrhosis or active hepatitis), to help wean them from mechanical ventilation. In order to determine the effects of theophylline treatment through a comparison of that treatment period with a period of non-treatment, we also looked at the patient data for the 16-month period immediately preceding the treatment period, that is, the period from March 2013 through June 2014, during which no patients received theophylline therapy. The medical records in question were retrospectively reviewed, and the patients were divided into 2 groups, a theophylline group (ie, those who were treated with theophylline) and a non-theophylline group (ie, those who were not treated with theophylline). The patients’ clinical data, including demographic factors, comorbidities, reason for prolonged mechanical ventilation, and number of mechanical ventilation days were recorded. For all the patients, Acute Physiology and Chronic Health Evaluation II (APACHE II) scores had been assessed within 24 h of admission. Global tests of respiratory muscle strength like measurements of the maximum inspiratory pressure (PImax)^[19]^ and rapid shallow breathing index (RSBI), which is the respiratory rate divided by tidal volume)^[20]^ were assessed at initial admission and then on a weekly basis thereafter until the last time before successful weaning or the last time before failed weaning and transfer to an respiratory care ward. In the theophylline group, the patients’ serum concentrations of theophylline were collected. The primary study goal was to compare the respiratory muscle strengths of the 2 groups by comparing their mean PImax and RSBI values. The secondary goal was to compare the 2 groups in terms of their weaning success rates and mean mechanical ventilation times. Weaning success was defined as the given patient being able to maintain breathing for 5 days without any mechanical ventilation support. For each patient, mechanical ventilation time was calculated as the amount of time from the initiation of mechanical ventilation until the end of the patient's time in the respiratory care center.

### 2.3 Statistical analysis

Continuous data were expressed as mean ± standard deviation, and the continuous data of the 2 study groups were compared using the Student *t* test and Mann-Whitney *U* test. Categorical data were expressed as frequencies and percentages, and these data were compared using the Chi-Square or Fisher exact test.^[21]^ All tests of significance were 2-tailed, and a *P* value <.05 was considered statistically significant. We relied on multiple linear regression to identify variables independently associated with post-theophylline treatment PImax and RSBI.^[22]^ Data were analyzed using SPSS software, version 19.0 (IBM).

## 3 Results

**Figure 1:**
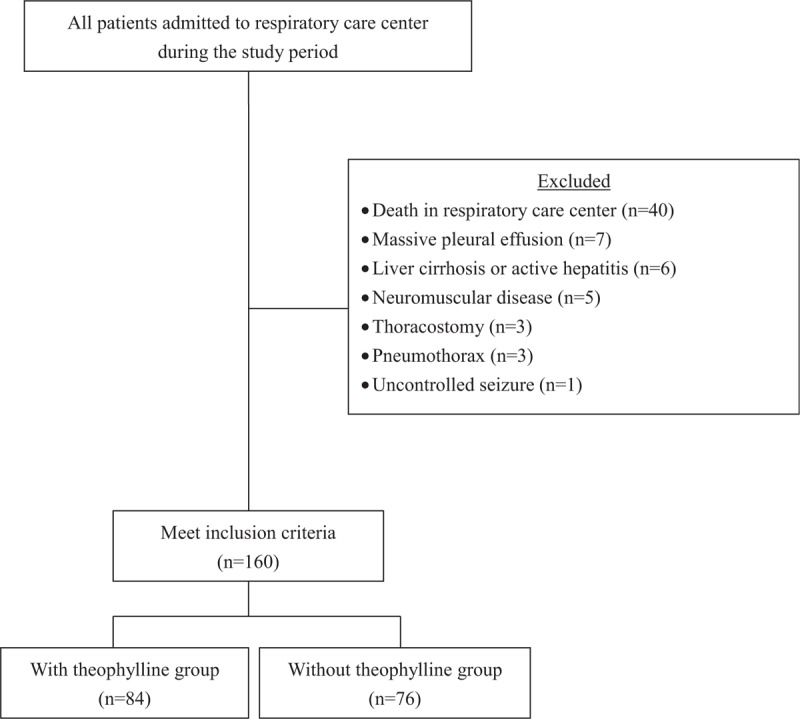
Subjects inclusion flowchart.

Of the 160 patients who fulfilled the inclusion criteria, 84 were included in the theophylline group and 76 were included in non-theophylline group (Fig. 1). The patient characteristics, demographics, and physiological and clinical variables are shown in Table 1. There were no between-group differences in those variables or in terms of comorbidities. However, the patients in the non-theophylline group had a significantly lower level of consciousness, as measured using a modified Glasgow Coma Scale (*P* = .013). The PImax and RSBI values indicated by the respiratory muscle strength tests conducted at initial admission were similar for the 2 groups. In addition, the original conditions necessitating prolonged mechanical ventilation were not significantly different between the groups.

**Table 1:**
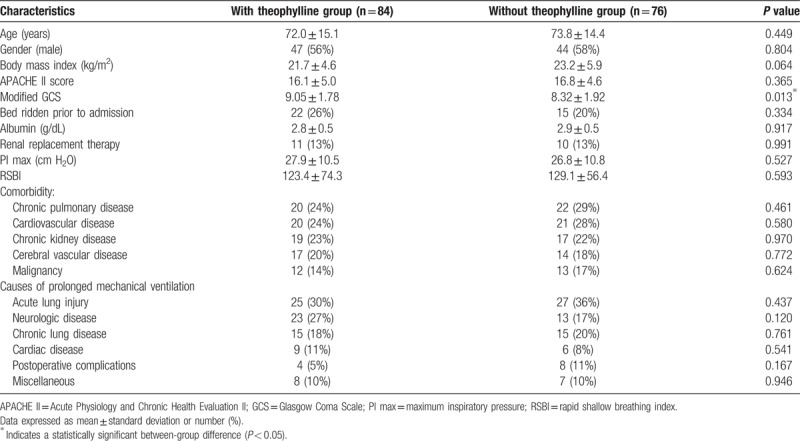
Clinical characteristics of prolonged mechanical ventilation patients treated with and without theophylline.

The theophylline-treated patients received 200 mg of aminophylline twice a day. The calculated dose of theophylline was thus 343 mg/d in those patients, who had a mean body weight of 60.7 ± 11.8 kg, and the mean treatment duration was 23 (9–34) days. The serum concentration of theophylline after the 3rd day of treatment, which was checked in 49 (58%) of the 84 patients, was 9.3 ± 3.3 μg/ml. No patient experienced any adverse effects of theophylline, such as nausea, vomiting, cardiac arrhythmias, or seizures. The primary outcomes, the last PImax and RSBI, were significantly better in the theophylline group than in the non-theophylline group, with a higher mean PImax (30.1 ± 9.7 cmH_2_O vs 26.9 ± 9.1 cmH_2_O; *P* = .034) and lower mean RSBI (107.0 ± 68.4 vs 131.4 ± 77.7; *P* = .036; Table 2). In terms of the percentage improvements to each respective patient's PImax and RSBI, they were significantly higher in the theophylline group than in the non-theophylline group (PImax: 20.1 ± 5.7% vs 3.2 ± 1.1%, *P* = .005; RSBI: 11.2 ± 3.0% vs. 2.7 ± 1.6%, *P* = .015; Table 2). We used a linear regression analysis to analyze the relationship between each of risk factors and the outcomes, that is, the last PImax and RSBI values. We only found that theophylline treatment had significant effect on the last PImax (*β* = 1.59, *P* < .05) and RSBI (β = −12.21, *P* < .05) values (Table 3). The secondary outcomes are shown in Table 2. The weaning success rate was higher in the theophylline group than in the non-theophylline group, but the difference was not statistically significant (78.6% vs 65.8%, *P* = .071). The mechanical ventilation time tended to be shorter in the theophylline group, but again, the difference was not statistically significant.

**Table 2:**
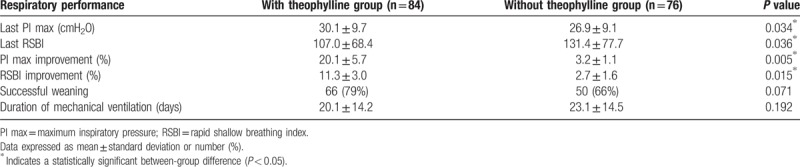
Comparison of respiratory performance of prolonged mechanical ventilation patients treated with and without theophylline.

**Table 3:**
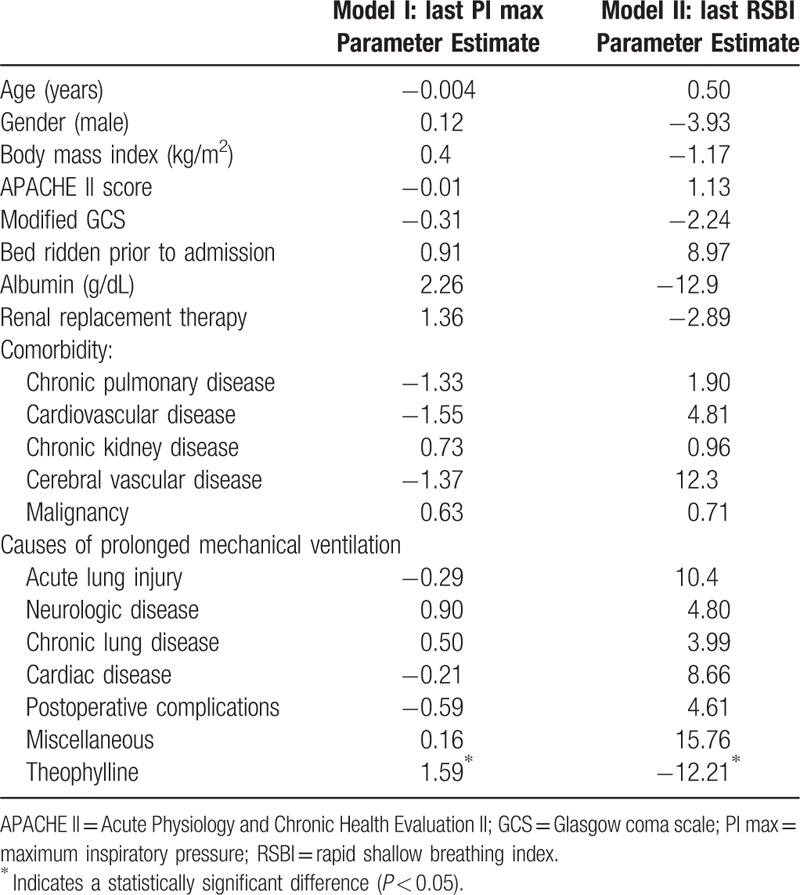
Linear regression analysis for PI max and RSBI.

## 4 Discussion

This study results showed that theophylline treatment significantly improved respiratory muscle function, in terms of improved PImax and RSBI, in patients with prolonged mechanical ventilation. The theophylline was also well tolerated, with no significant adverse reactions among the study patients. To our knowledge, this is little study to evaluate the efficacy and safety of theophylline in weaning patients from prolonged mechanical ventilation.

Recent studies have suggested that the prevalence of ventilator-induced diaphragmatic weakness is very high and that this harm can even occur on the first day of mechanical ventilation in an ICU.^[23]^ That said, the degree of diaphragmatic weakness, injury, and atrophy is significantly correlated with the duration of ventilator support.^[9]^ According to previous studies, prolonged mechanical ventilation patients who experience difficulty in being weaned from the mechanical ventilation should have a high prevalence of diaphragmatic weakness and function loss.

Mechanical ventilation-induced diaphragmatic unloading is associated with oxidative stress in the diaphragm due to the production of reactive oxygen species, with this problem potentially occurring even within 3 to 6 h after the initiation of mechanical ventilation.^[24]^ Animal studies have revealed that the activities of the enzymes nicotinamide adenine dinucleotide phosphate oxidase and xanthine oxidase, being potential sources of reactive oxygen species, are significantly elevated in the diaphragm after prolonged mechanical ventilation.^[25,26]^ Theophylline inhibits xanthine oxidase activity and therefore may protect the diaphragm against mechanical ventilation-induced oxidative stress and contractile dysfunction. Furthermore, in studies of patients with chronic obstructive pulmonary disease, theophylline has been found to have a potent and long-lasting effect in terms of increasing the strength of and suppressing fatigue in the diaphragm.^[27]^ Several other studies have also suggested that theophylline increases diaphragmatic contractility and reduces diaphragmatic fatigue.^[28,29]^ Our results are consistent with these previous studies and suggest that theophylline may benefit prolonged mechanical ventilation patients by improving their diaphragmatic function.

In a recent study of ICU patients that used ultrasonographic examinations for the assessment of diaphragmatic motion, theophylline was found to significantly improve the diaphragmatic movements of patients with VIDD, whereas it did not affect the diaphragmatic movements of patients without VIDD.^[10]^ In that study, all the patients included had been treated with mechanical ventilation for at least 72 hours, while cases involving prolonged weaning were excluded. Besides, they examined diaphragmatic motion by ultrasound. They did not study weaning profile such as PImax and RSBI to assess whole respiratory muscle strength, as they are global measures of it.^[19,20]^ In our study, the patients all experienced weaning difficulty and had used mechanical ventilation for at least 21 days. According to previous studies, patients with prolonged mechanical ventilation should have a high prevalence of VIDD.^[9,23]^ However, theophylline has been shown to have inotropic effects on diaphragms, and the results of this study demonstrated the beneficial effects of theophylline on PImax and RSBI when attempting to wean patients from prolonged mechanical ventilation. According to our results, the weaning success rate was higher for the theophylline group than for the non-theophylline group, although not significantly so. One possible explanation for this finding is that the weaning of prolonged mechanical ventilation patients from mechanical ventilation is a difficult and multi-factorial process, one that does not rely solely on respiratory muscle strength.

Most patients in the respiratory care center receiving mechanical ventilation are fed via a nasogastric tube. In our hospital, the theophylline preparation typically used is a sustained-release preparation that cannot be ground up for nasogastric tube administration. So, we treated the patients in this study with aminophylline, which contains 85.7% anhydrous theophylline. The calculated dose of theophylline was 343 mg/d, with the mean checked serum concentration being 9.3 μg/ml, which is near the lower limit of a therapeutic dose (mean serum concentration ≥10 μg/ml) as assessed by previous studies.^[27,30]^ That said, it is interesting to note that in the previously mentioned study, even low doses of theophylline resulting in a mean serum concentration of 4.6 μg/ml improved diaphragmatic movements.^[10]^ Relatedly, it has been reported that even low concentrations of theophylline (<5 μg/ml) can restore and increase histone deacetylase-2 activity, thus reducing oxidative stress in the diaphragm.^[31]^ That said, given that the current study involved patients with different characteristics than the patients in those earlier studies, lower doses and the dose-response relationships of theophylline require further study, especially with regard to possible adverse effects.

The present study had many limitations. First, the study was retrospective and the design might be not rigorous enough. So, there might latent bias existed, although we’ve tried our best to avoid it. Second, we measured PImax and RSBI to assess respiratory muscle strength, as they are global measures of it. However, these 2 measures may lack specificity. As such, further studies are warranted and should, if possible, seek to avoid the limitations of the present study.

## 5 Conclusions

In conclusion, the results of the current study indicated that theophylline improved diaphragmatic strength in patients with prolonged mechanical ventilation and may facilitate ventilator weaning. Further larger prospective studies are needed, however, to confirm these results.

## Author contributions

**Conceptualization:** Kuo-Chin Kao.

**Data curation:** Teng-Jen Yu, Yu-Chih Liu, Chien-Min Chu, and Han-Chung Hu.

**Formal analysis:** Teng-Jen Yu, Yu-Chih Liu, Chien-Min Chu, and Han-Chung Hu.

**Investigation:** Teng-Jen Yu, Yu-Chih Liu, Chien-Min Chu, Han-Chung Hu, and Kuo-Chin Kao.

**Methodology:** Yu-Chih Liu and Kuo-Chin Kao.

**Supervision:** Kuo-Chin Kao.

**Validation:** Yu-Chih Liu.

**Writing – original draft:** Teng-Jen Yu.

**Writing – review & editing:** Kuo-Chin Kao.
